# Voltage-gated calcium channels: Their discovery, function and importance as drug targets

**DOI:** 10.1177/2398212818794805

**Published:** 2018-10-02

**Authors:** Annette C. Dolphin

**Affiliations:** Department of Neuroscience, Physiology & Pharmacology, University College London, London, UK

**Keywords:** Calcium, channel, voltage, second messenger, neuron, heart

## Abstract

This review will first describe the importance of Ca^2+^ entry for function of excitable cells, and the subsequent discovery of voltage-activated calcium conductances in these cells. This finding was rapidly followed by the identification of multiple subtypes of calcium conductance in different tissues. These were initially termed low- and high-voltage activated currents, but were then further subdivided into L-, N-, PQ-, R- and T-type calcium currents on the basis of differing pharmacology, voltage-dependent and kinetic properties, and single channel conductance. Purification of skeletal muscle calcium channels allowed the molecular identification of the pore-forming and auxiliary α_2_δ, β and ϒ subunits present in these calcium channel complexes. These advances then led to the cloning of the different subunits, which permitted molecular characterisation, to match the cloned channels with physiological function. Studies with knockout and other mutant mice then allowed further investigation of physiological and pathophysiological roles of calcium channels. In terms of pharmacology, cardiovascular L-type channels are targets for the widely used antihypertensive 1,4-dihydropyridines and other calcium channel blockers, N-type channels are a drug target in pain, and α_2_δ-1 is the therapeutic target of the gabapentinoid drugs, used in neuropathic pain. Recent structural advances have allowed a deeper understanding of Ca^2+^ permeation through the channel pore and the structure of both the pore-forming and auxiliary subunits. Voltage-gated calcium channels are subject to multiple pathways of modulation by G-protein and second messenger regulation. Furthermore, their trafficking pathways, subcellular localisation and functional specificity are the subjects of active investigation.

## Introduction: the importance of Ca^2+^ entry for function of excitable cells

**Figure 1.**
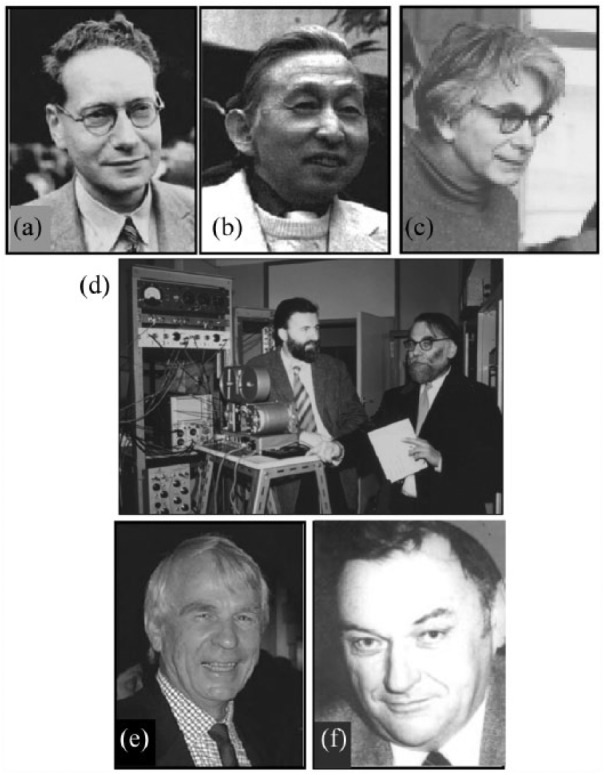
Some key figures in the early discovery of calcium channels and their pharmacology: (a) Bernard Katz, (b) Susumu Hagiwara, (c) Paul Fatt, (d) Bernard Ginsborg (right) demonstrating equipment similar to that used to record crustacean muscle action potentials, (e) Harald Reuter and (f) Albrecht Fleckenstein. (c) is taken from a photograph (1978) by Martin Rosenberg, the Physiological Society; reproduced with permission; (a), (b) and (f) are reproduced from with permission from Richard W. Tsien (Barrett and Tsien, 2004); (d) is reproduced with permission from Bernard Ginsborg, who died this year (1925–2018).

It has been clear from the time of Sydney Ringer, working at University College London, that calcium ions (Ca^2+^) are essential for heart muscle contraction (Ringer, 1883). However, the paramount importance of Na^+^ and K^+^ for the activation and inactivation underlying action potential generation led to Ca^2+^ permeation being little studied for many years. In the 1950’s Paul Fatt, working at University College London with both Katz and Ginsborg, found that Ca^2+^ supports action potential-like spikes in crustacean muscle (Fatt and Ginsborg, 1958; Fatt and Katz, 1953), and this was also found to be true in barnacle muscle (Hagiwara and Takahashi, 1967). When it was also identified that Ca^2+^ was essential for neurotransmitter release (Katz and Miledi, 1967), it became clear that calcium ion entry through membranes was key to many important processes in nerves as well as muscle. These key players in the field are pictured in Figure 1(a)–(d).

## Identification of multiple subtypes of calcium channel

A major contribution to the understanding of calcium channel function then came from Harald Reuter (Figure 1(e)), who showed, using microelectrodes, that calcium currents were present in voltage-clamped cardiac Purkinje fibres (Reuter, 1967). The advent of the gigaseal patch-clamp method for recording currents through the membrane of single cells (Hamill et al., 1981) then allowed single calcium channels to be resolved (Fenwick et al., 1982).

The discovery and use of verapamil, and the 1,4-dihydropyridines (DHPs) including nifedipine, as antihypertensive drugs represented a very important advance (Fleckenstein, 1983) (Figure 1(f)). Their target was found to be inhibition of cardiovascular calcium channels (Lee and Tsien, 1983); thus, the term calcium channel blocker or antagonist was coined. Related drugs were found to have agonist effects (Schramm et al., 1983), to increase cardiac calcium conductance and prolong single channel openings (Hess et al., 1984). Both the agonist and antagonist drugs gave researchers important tools to dissect calcium channel function in a variety of tissues.

The first suggestion that there was more than one component to calcium currents in different tissues came from the group of Hagiwara et al. (1975), followed by evidence of low threshold Ca^2+^ spikes in mammalian central neurons (Llinás and Yarom, 1981), and distinct low voltage-activated currents in peripheral dorsal root ganglion neurons (Carbone and Lux, 1984; Fedulova et al., 1985; Nilius et al., 1985).

## Identification of N-, P- and R-type calcium currents as distinct from L-type channels

**Figure 2.**
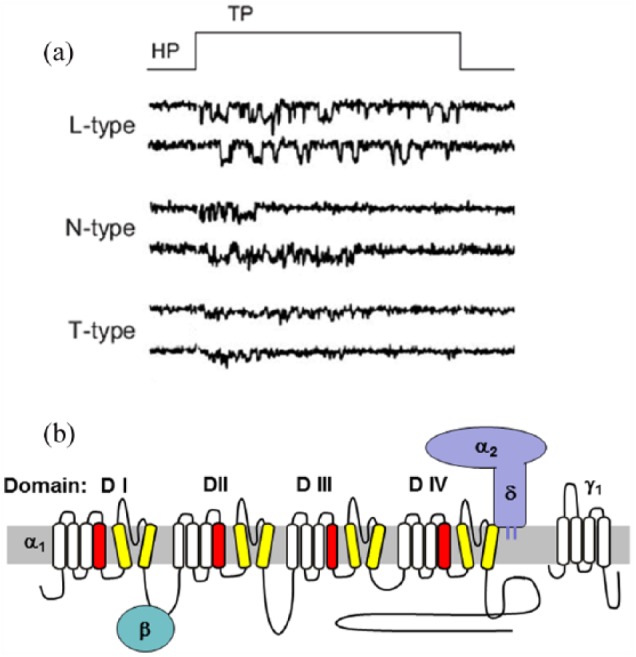
Single calcium channels with different properties, and topology of the channels. (a) Identification of a third component of voltage-gated calcium channels (N-type) from the biophysical properties of single channel currents observed in cell-attached patches on dorsal root ganglion neurons. Redrawn from Nowycky et al. (1985). TP: test potential; HP: holding potential. Reproduced with thanks to Richard W. Tsien. (b) Diagram of α_1_ subunit topology and calcium channel subunit structure, also showing α_2_δ (purple) and β (blue). ϒ_1_ is only present in skeletal muscle calcium channel complexes. S4 voltage sensors in each α_1_ domain are represented by red transmembrane segments. Yellow denotes S5 and S6 pore transmembrane segments in each domain.

In dorsal root ganglion (DRG) neurons, it was then found that there were three calcium current components. The DHP-sensitive current was designated L-type (for long-lasting, which also had a large singe channel conductance) and the low-voltage activated component was termed T (for transient, which also had a Tiny single channel conductance). A third component, which was high-voltage activated but DHP-insensitive, was termed N-type (neither L nor T, and also exclusively Neuronal) (Fox et al., 1987; Nowycky et al., 1985) (Figure 2(a)). A blocker of this component was not long in appearing. A toxin component from the marine snail *Conus geographus*, ω-conotoxin GVIA, first thought to block both neuronal L- and N-type calcium currents (McCleskey et al., 1987), was later found to be highly selective for N-type channels (Boland et al., 1994; Plummer et al., 1989). Using this pharmacological blocker, N-type calcium currents were then shown to play a key role in neurotransmitter release (Hirning et al., 1988).

The importance of pharmacological tools in the discovery of calcium channel subtypes became even more evident when it was found that the calcium current in Purkinje neurons was not blocked by DHPs or by ω-conotoxin GVIA. This current was called P-type (for Purkinje) (Llinás et al., 1989). The same group used a polyamine toxin (FTX) from the American funnel web spider to block Purkinje cell Ca^2+^ currents, but FTX was not particularly selective for P-type channels, whereas a peptide toxin component from the same spider (ω-agatoxin IVA) was more selective, blocking fully the calcium current in Purkinje neurons (Mintz et al., 1992). This toxin also inhibited a component of the calcium current in cerebellar granule cells (Pearson et al., 1995; Randall and Tsien, 1995), which was initially termed Q-type as it had different biophysical properties from that in Purkinje neurons (Randall and Tsien, 1995); however, these are usually now called PQ currents. That study also identified an additional resistant current component in cerebellar granule cells which was designated R-type (Randall and Tsien, 1995), and a similar novel component was also identified in bullfrog sympathetic neurons (Elmslie et al., 1994). A tarantula toxin, SNX-482, was identified to block this component (Newcomb et al., 1998), but it has subsequently been found also to block other channels (Kimm and Bean, 2014), complicating interpretation of physiological experiments using SNX-482.

## Purification and molecular identification of the calcium channel subtypes

Receptors for the DHP calcium antagonists were identified using [^3^H]-nitrendipine to guide purification. They were found to be highly concentrated in the t-tubules of skeletal muscle (Fosset et al., 1983), where they were shown to be responsible for charge movement and excitation-contraction coupling (Rios and Brum, 1987). Purification studies identified the skeletal muscle DHP receptor to be a complex of five polypeptides in approximately equal amounts, and therefore considered to be subunits. They were termed, in decreasing order of size, the α_1_, α_2_, β, ϒ and δ subunits (Hosey et al., 1987; Takahashi et al., 1987). The 175 kDa α_1_ subunit was tentatively identified as the pore-forming subunit of the channel, since it bound radiolabelled DHP. The associated proteins were termed auxiliary or accessory subunits.

Peptide sequence from the purified DHP receptor protein enabled the identification of probes and subsequent cloning of the skeletal muscle calcium channel (Ellis et al., 1988; Tanabe et al., 1987). The hydropathy plot indicated that it was a 24 transmembrane spanning protein, with four homologous repeated domains joined by intracellular linkers, similar to recently cloned voltage-gated Na^+^ channel (Noda et al., 1984) (Figure 2(b)). This protein was termed α_1_S (for skeletal muscle) and was indisputably shown to encode a calcium channel by injection of its cDNA into dysgenic skeletal myotubes which lack the mRNA for α_1_S (Tanabe et al., 1988). This restored excitation–contraction coupling, as well as the very slow calcium current observed in native skeletal muscle.

The cardiac L-type calcium channel, termed α_1_C, was then cloned by homology with α_1_S (Mikami et al., 1989). Prior to this time, the unique permeation selectivity of the voltage-gated calcium channels for Ca^2+^ had already been attributed to high affinity Ca^2+^ binding in the pore of the channel (Hess and Tsien, 1984), and this was borne out by identification of key glutamate residues in the pore ‘P loops’ (Yang et al., 1993), whose acidic side chains were surmised to participate in Ca (α-amino-3-hydroxy-5-methyl-4-isoxazolepropionic acid)^2+^ binding and permeation.

Several brain calcium channels were then cloned and identified to encode P- and N-type channels (Mori et al., 1991; Snutch et al., 1990; Starr et al., 1991). These were termed α_1_A and α_1_B, respectively. Another channel was cloned and dubbed α_1_E (Soong et al., 1993). It was first classified as a low-voltage activated T-type channel, but it soon became clear that it did not have the expected properties, and it is now considered to encode R-type channels. Genes for three T-type channels were later cloned by Perez-Reyes and colleagues (Cribbs et al., 1998; Lee et al., 1999; Perez-Reyes et al., 1998). These were termed α_1_G, H and I. In addition, two further L-type channels were identified. The first, cloned from brain, was called α_1_D (Williams et al., 1992) and was shown to have distinctive biophysical properties, being lower voltage-activated than α_1_C (Koschak et al., 2001; Xu and Lipscombe, 2001). Finally, a fourth L-type channel was identified because of its role in a genetic form of night blindness (Bech et al., 1998; Strom et al., 1998), and this was also shown to have properties distinguishing it from the other L-type channels (Koschak et al., 2003).

Following the cloning and initial study of all the calcium channel α_1_ subunits identified in the mammalian genome, a rationalised nomenclature was adopted in 2000, grouping the α_1_ subunits into Ca_V_1 (L-type), Ca_V_2 (non-L-type) and Ca_V_3 (T-type) (Ertel et al., 2000) (Table 1). Since that time the distinctive properties of multiple splice variants of these channels have also been recognised.

**Table 1.** Subtypes of calcium channel.

Activation voltage	Functional nomenclature	Channel α_1_ subunit	Ca_V_ nomenclature	Main function
(High)	L	a_1_S	1.1	Skeletal muscle voltage sensor
High	L	a_1_C	1.2	Cardiac, smooth muscle function
Medium	L	a_1_D	1.3	Hearing, sino-atrial node function
Medium	L	a_1_F	1.4	Retinal neurotransmission
High	PQ	a_1_A	2.1	Synaptic transmission in CNS, motor nerves and elsewhere
High	N	a_1_B	2.2	Synaptic transmission in PNS (and CNS, especially early in development)
Medium	R	a_1_E	2.3	Present in some neurons and synapses
Low	T	a_1_G	3.1	Neuronal excitability, pacemaker activity, subthreshold oscillations
Low	T	a_1_H	3.2	
Low	T	a_1_I	3.3	

PNS: peripheral nervous system; CNS: central nervous system.

## Importance of auxiliary subunits

The auxiliary β subunit from skeletal muscle was the first to be cloned (Ruth et al., 1989) (Figure 2(b)). It was subsequently termed β_1a_, after three further isoforms (β_2_, β_3_ and β_4_) as well as multiple splice variants were identified by homology. β_1b_ is the non-muscle splice variant of β_1_ (Pragnell et al., 1991), and β_2a_ is a palmitoylated β_2_ splice variant, giving it distinctive properties (Qin et al., 1998). The importance of these β subunits to the expression of the Ca_V_1 and Ca_V_2 channels was clear from antisense knockdown studies in native tissues and early expression studies (Berrow et al., 1995; Qin et al., 1998). In contrast, the Ca_V_3 channels do not appear to have any obligate auxiliary subunits.

When the auxiliary α_2_δ subunit was cloned, it was realised that α_2_ and δ are encoded by the same gene and form a pre-protein, which is then proteolytically cleaved, but the α_2_ and δ proteins remain associated by pre-formed disulphide bonding (De Jongh et al., 1990; Jay et al., 1991). Its proteolytic cleavage has recently been shown to be essential for α_2_δ function (Kadurin et al., 2016). The skeletal muscle α_2_δ was subsequently termed α_2_δ-1, when three further mammalian isoforms were identified: α_2_δ-2 (Barclay et al., 2001; Gao et al., 2000), α_2_δ-3 and α_2_δ-4 (Qin et al., 2002). The muscle α_2_δ subunit was first described as a transmembrane protein, but they have subsequently been shown to be glycosyl-phosphatidylinositol (GPI)-anchored into the outer leaflet of the plasma membrane (Davies et al., 2010) (Figure 2(b)). The α_2_δ subunit was predicted to contain a von Willebrand factor A (VWA) domain, which was found to be essential for trafficking, both of α_2_δ itself, and for its effect on the α_1_ subunits (Canti et al., 2005; Cassidy et al., 2014; Hoppa et al., 2012).

The skeletal muscle calcium channel complex also contains a ϒ subunit, now called ϒ_1_ (Takahashi et al., 1987) (Figure 2(b)), but ϒ is not associated with other calcium channels, and further members of this ‘ϒ subunit’ family are now known to be trafficking proteins that modulate the function of AMPA (α-amino-3-hydroxy-5-methyl-4-isoxazolepropionic acid) glutamate receptors, rather than voltage-gated calcium channel subunits (Tomita et al., 2003). The roles of the different calcium channel auxiliary subunits have been more extensively reviewed recently (Dolphin, 2012).

## Elucidation of physiological channel function from knockout mouse studies and genetic mutations

Several spontaneously arising mouse loss-of-function mutants were identified which gave important clues as to the function of the channel subunits. This was particularly true for Ca_V_2.1, β4 and α_2_δ-2 which are strongly expressed in cerebellum, and whose mutation produced obvious ataxias (Barclay et al., 2001; Burgess et al., 1997; Fletcher et al., 1996). Subsequent targeted knockouts gave similar phenotypes. A surprise came with the knockout of Ca_V_1.3, both in mice and in a homozygous human mutation, in whom the main phenotype was deafness and sino-atrial node dysfunction (Baig et al., 2011). Furthermore Ca_V_1.4 was identified from its role in a retinal disease (Bech-Hansen et al., 1998; Strom et al., 1998), and the knockout mouse has a similar phenotype (Mansergh et al., 2005). Knockout of Ca_V_2.2 resulted in a diminution of neuropathic pain responses, reinforcing its importance in primary afferent neurotransmission (Saegusa et al., 2001). Similarly, α_2_δ-1 knockout delayed the onset of mechanical hyperalgesia following neuropathic injury (Patel et al., 2013) and α_2_δ-3 has a role in hearing (Pirone et al., 2014), and in the central control of pain (Neely et al., 2010).

## Structural studies

The first components of the calcium channel complex to be amenable to structural studies were the β subunits, which contain two conserved interacting domains (SH3 and guanylate kinase-like), the latter binding to the linker between domains I and II of the channels (Chen et al., 2004; Opatowsky et al., 2004; Pragnell et al., 1994; Richards et al., 2004; Van Petegem et al., 2004).

The first crystal structure for a calcium-selective voltage-gated channel was obtained using a mutant form of a bacterial sodium channel homolog, Na_V_Ab, a single domain channel which forms homo-tetramers (Payandeh et al., 2011). This was mutated so that the pore became Ca^2+^-selective, forming Ca_V_Ab. This structure has provided multiple insights, including confirmation of the Ca^2+^ permeation process (Tang et al., 2014). Remarkably, this channel was sensitive to calcium channel antagonists, yielding further important insight into the binding and mechanism of action of these drugs (Tang et al., 2016). For mammalian calcium channel complexes, although low-resolution single particle electron microscopic structures were published previously (Serysheva et al., 2002; Walsh et al., 2009; Wolf et al., 2003), major advances in cryo-electron microscopy were needed before a detailed structure of the skeletal muscle calcium channel was produced, very beautifully elucidating details of the pore and the subunit arrangement (Wu et al., 2016). GPI-anchoring of α_2_δ (Davies et al., 2010), and interaction of the α1 subunit with the VWA and Cache domains (which have similarity to bacterial chemotaxis domains) of α_2_δ (Canti et al., 2005; Cassidy et al., 2014), were confirmed in the structure (Wu et al., 2016).

## Calcium channel modulation

Only two canonical second messenger modulation pathways will be considered here, for reasons of space: inhibitory modulation of neuronal calcium channels by G-proteins, and cyclic AMP-dependent phosphorylation, mediating enhancement of L-type channels. Many other pathways also deserve mention, including Ca^2+^-calmodulin control of Ca^2+^-dependent inactivation and facilitation of L-type and P-type channels, studied extensively by the late David Yue (Dick et al., 2008; Peterson et al., 1999).

### G-protein modulation

**Figure 3.**
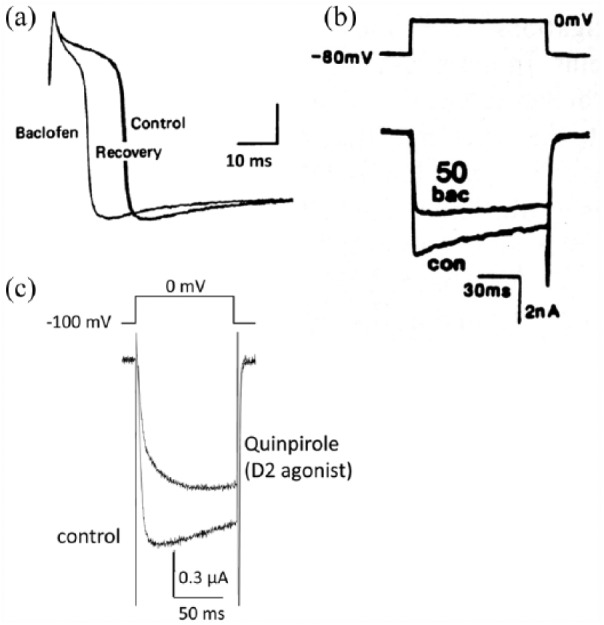
Inhibitory G-protein modulation of neuronal calcium channels. (a) Action potential (prolonged by K^+^ channel blockade), recorded from dorsal root ganglion neuron, showing the control, inhibition by the GABA-B agonist baclofen (100 μM) and recovery (from Figure 7 of Dolphin et al. (1986)). (b) Calcium channel currents recorded from dorsal root ganglion neuron, showing inhibition by baclofen (bac, 50 μM) (Dolphin et al., 1989). (c) Calcium channel currents recorded from *Xenopus laevis* oocytes injected with Ca_V_2.2/β3/α_2_δ-1 and the dopamine D2 receptor, showing inhibition by the D2 agonist quinpirole (100 nM) (replotted from Figure 2(e) of Canti et al. (2000).

Voltage-dependent activation of neuronal calcium channels is required for neurotransmitter release, and this process can be inhibited by a range of modulatory neurotransmitters coupled to seven-transmembrane receptors (Dolphin, 1982; Jessell and Iversen, 1977; Peng and Frank, 1989), leading to the view that inhibitory modulation of the calcium channel-mediated component of the presynaptic action potential underpins receptor-mediated presynaptic inhibition (Dolphin et al., 1986; Dunlap and Fischbach, 1978; Ikeda and Schofield, 1989) (Figure 3(a)). Modulation of neurotransmitter release was found to be mediated by a pertussis toxin-sensitive GTP-binding protein, of the G_i_/G_o_ family (Dolphin and Prestwich, 1985). The inhibitory modulation of neuronal calcium currents was subsequently also identified to involve these G-proteins (Scott and Dolphin, 1986; Holz et al., 1986) (Figure 3(c)). Using both native and cloned Ca_V_2 channels, the modulation was subsequently shown to be a direct membrane-delimited effect of Gβϒ subunits (Herlitze et al., 1996; Ikeda, 1996), mediated by the channel I-II linker (Bourinet et al., 1996) and its intracellular N-terminus (Page et al., 1998). The characteristic voltage-dependence of the inhibition, which means that inhibition is lost with large or repeated depolarisations, was shown to require participation of the calcium channel β subunit (Meir et al., 2000).

### Cyclic AMP-dependent phosphorylation

Another key example of second messenger modulation is provided by L-type calcium channels, which are potentiated by β-adrenergic receptor activation, via a cyclic AMP-dependent mechanism (Cachelin et al., 1983; Reuter, 1983). In heart, this effect is mediated by β1-adrenergic receptors and forms one of the main components of the fight-or-flight response. However, it has been difficult to reproduce when cloned Ca_V_1.2 calcium channels are expressed, for example, in HEK-293 cells, suggesting it is more complex than simple channel phosphorylation, and indeed, the role of the several protein kinase A substrate serines in cardiac Ca_V_1.2 function is still being determined (Lemke et al., 2008; Yang et al., 2016). Furthermore, the response to β-adrenergic stimulation may involve a proteolytically cleaved C-terminal fragment of the endogenous Ca_V_1.2 channels (Fu et al., 2013; Fuller et al., 2010). Perhaps surprisingly, there appears to be a somewhat different basis for the spatially restricted stimulation observed in hippocampal neurons following activation by β2-adrenergic receptors of neuronal Ca_V_1.2 channels (Qian et al., 2017).

## Future research

The selective pharmacology that has been so important for dissecting out the functions of different calcium channels is still incomplete. Although a selective inhibitor of the T-type calcium channels exists (Dreyfus et al., 2010), it does not differentiate between the Ca_V_3 channels. Similarly, there are currently no selective inhibitors of the different Ca_V_1 channels. Such inhibitors that would be able to differentiate between these very similar channels could have important therapeutic possibilities. For example, selective inhibition of Ca_V_3.2 could be of therapeutic benefit in certain types of pain (Marger et al., 2011), and selective inhibitors of Ca_V_1.3 have potential for therapeutic use in Parkinson’s disease and other disorders (Striessnig et al., 2015). Furthermore, although ω-conotoxin GVIA is a selective blocker of N-type channels and a related compound is licenced for use intrathecally in some chronic pain conditions (Miljanich, 2004), no small molecule inhibitors of N-type channels have yet been shown to be effective in clinical trials for chronic pain.

Future challenges include a full understanding of how particular calcium channels are trafficked into precise subcellular domains, for example, how some channels are targeted to dendrites (Hall et al., 2013), while others are directed to presynaptic active zones to mediate neurotransmitter release (Kaeser et al., 2011). Furthermore, calcium channels have been found to interact, directly or indirectly, with multiple scaffolding proteins, ion channels and second messenger pathways (Müller et al., 2010), but how these are organised and function together remains to be elucidated. Related to this, the pathways for intracellular Ca^2+^ signalling to the nucleus and the selectivity for L-type Ca^2+^ channels in neurons are still being revealed (Cohen et al., 2015; Wheeler et al., 2012).
